# Effects of Acupuncture on Neurological Disease in Clinical- and Animal-Based Research

**DOI:** 10.3389/fnint.2019.00047

**Published:** 2019-08-30

**Authors:** Xiangyu Guo, Tao Ma

**Affiliations:** Dongfang Hospital, Beijing University of Chinese Medicine, Beijing, China

**Keywords:** acupuncture, neurological disease, Alzheimer’s disease, Parkinson’s disease, clinical trial, animal model

## Abstract

Neurological disease, including Alzheimer’s disease (AD), Parkinson’s disease (PD), which were caused by abnormalities in the nervous system involves the accumulation of false proteins, neurotransmitter abnormalities, neuronal apoptosis, etc. As an alternative supplementary medicine (ASM), acupuncture plays an important role in the treatment of neurological diseases. In this review article, we summarized the current evidence for the treatment efficacy of acupuncture in AD and PD from the perspective of clinical trials and animal model. Acupuncture can inhibit the accumulation of toxic proteins in neurological diseases, modulate energy supply based on glucose metabolism, depress neuronal apoptosis, etc., and exert a wide range of neuroprotective effects.

## Introduction

Neurological diseases are disorders of the nervous system. Neurological diseases can manifest a series of symptoms due to abnormalities in nerve structure, biochemistry or electrophysiology in the brain, spinal cord or other nerves. Most of them are characterized by progressive neurodegeneration and injury closely related to aging, including Alzheimer’s disease (AD), Parkinson’s disease (PD), amyotrophic lateral sclerosis (ALS), Frontotemporal dementia (FTD), et cetera (Devereux et al., 2004). Aging is a common risk factor for all of these diseases. With the aging of the world’s population, the incidence of these devastating diseases is increasing, which poses a greater threat to the world’s human health and increases enormous economic burden on patients, families, and social communities (Hurd et al., 2013). Although the pathogenesis of neurodegenerative diseases varies, they still share many common pathogenic features and mechanisms, such as misfolded protein aggregation, neuronal apoptosis, and synaptic loss, neurotransmitter abnormalities, et cetera (Gan et al., 2018).

Acupuncture has been used as an alternative supplementary medicine (ASM) in China, Korea, Japan and other East Asian countries for hundreds of years (Freedman and Bierwirth, 2018). Acupuncture, as a traditional alternative medical treatment, has a significant clinical efficacy on the treatment of neurological diseases, especially neurodegenerative diseases (Shin et al., 2017; Freedman and Bierwirth, 2018). The therapeutic effect of acupuncture treatment is to insert a needle into specific acupoints on the body surface, while manually rotating or applying electric pulse stimulation (Cai and Shen, 2018). Stimulation of peripheral nerves by acupuncture can recover and repair a variety of neurological diseases (Lee et al., 2007; Park and Lee, 2010; Li X. et al., 2017). This review article will summarize the research progress of clinical and animal-based on acupuncture treatment of neurological diseases in recent years, especially for AD and PD.

## Alzheimer’s Disease

AD is currently the leading cause of dementia, accounting for 60%–80% of the total number of dementia cases (Alzheimer’s Association, 2016). AD is mainly characterized by progressive memory disorders, aphasia, agnosia, apraxia and personality degeneration. The most important risk factor for AD is aging. There are currently no drugs that can prevent or reverse the progression of AD. FDA-approved drugs for the treatment of AD can only ameliorate the symptoms of AD and have many adverse effects. As a consequence, a large number of AD patients treat memory impairment and improve their quality of life through ASM. As one of the most popular ASMs, acupuncture is widely used in AD treatment. Clinical evidences have shown that acupuncture can significantly improve the language and motor function and ameliorate the emotional and cognitive impairments of AD patients (Chen, 1992; Zhou et al., 2017; Kan et al., 2018).

### Clinical Research on Acupuncture Treatment for AD

In a Chinese trial, 38 AD subjects were received acupuncture at specific acupoints, and 16 subjects developed symptoms after treatment (Chen, 1992). In a meta-analysis of 10 randomized controlled trials (RCTs) of acupuncture treatment of AD involving 585 subjects, six trials showed that acupuncture has a better effect than drugs at improving Mini Mental State Examination (MMSE) scale scores of AD patients, and mean differences (MD) is 1.05 with 95% confidence intervals (CIs) 0.16–1.93; evidence from the pooled results of three trials showed that acupuncture combined with donepezil is more effective than donepezil alone in improving MMSE scale scores in AD patients (MD 2.37, 95% CI 1.53–3.21; Zhou et al., 2015). As for the safety of acupuncture in the treatment of AD, the analysis indicated that there were only two acupuncture-related adverse reactions in 141 clinical trials; only 7 of the 3,416 subjects had acupuncture-related adverse reactions, so the study concluded that these reactions are within the allowable range and are not severe (Zhou et al., 2015).

In addition, acupuncture is valued especially in the treatment of mild-to-moderate AD. In a large clinical study, 87 patients with mild-to-moderate AD received monotherapy, three times acupunctures per week or daily donepezil for 12 weeks. The results showed that as compared to the donepezil group, the AD Assessment Scale-Cognitive (ADAS-cog) scores were significantly lower in the acupuncture group at 28 weeks; the mean values of Clinician’s Interview Based Impression of Change-Plus (CIBIC-Plus) were significantly decreased in the same group at weeks 10 and 28, and cognitive function improvement can be observed at the end of acupuncture treatment (weeks 12; Jia Y. et al., 2017). In another small-scale clinical trial, eight patients with mild-to-moderate AD received acupuncture treatment. Each patient was treated for 30 days and given in a 7-day treatment with a 3-day break. By measuring the verbal orientation, motor coordination and overall score in the patient’s MMSE score, acupuncture can significantly improve the patient’s cognitive level and produced a remarkable clinical improvement from baseline on the Traditional Chinese Medicine Symptoms Checklist for AD (Kao et al., 2000; Tu et al., 2019). In the treatment of patients with mild cognitive impairment (MCI), a small-scale clinical study based on functional magnetic resonance imaging (fMRI) revealed the relationship between *de qi* sensations produced by different acupuncture depths during acupuncture treatment and evaluated their impacts on cognition on the whole brain network. The results show that deep acupuncture can induce a stronger and broader de qi sensation than superficial acupuncture, and can enhance the centrality of nodes in the cerebral regions related to cognitive impairment in MCI patients (Bai et al., 2013; Tu et al., 2019).

### Animal-Based Research on Acupuncture Treatment for AD

Studies have shown that acupuncture can play a therapeutic role by modulating multiple aspects of AD pathological processes in animal models, including accumulation of amyloid-β (Aβ), inhibition of neuronal apoptosis, and regulation of glucose-related energy metabolism, et cetera (Cheng et al., 2008; Li et al., 2012; Lee et al., 2014; Guo et al., 2015).

#### Amyloid-β Protein

The abnormal accumulation of Aβ protein in the brain and the formation of senile plaques are key typical pathological features of AD. Acupuncture can down-regulate beta-secretase 1 (BACE1), a key enzyme affecting Aβ production, by stimulating GV14 (Deachu) and BL23 (Sinsu) of senescence-accelerated mouse prone 8 (SAMP8) mice, a rapidly aging mouse that mimics AD lesions (Dong et al., 2015). On the other hand, activation of AMP-activated protein kinase (AMPK) can inhibit the accumulation of Aβ. Acupuncture could activate AMPK by stimulating GV14 and BL23 in SAMP8 mice, thereby inhibiting Aβ production (Dong et al., 2015). Furthermore, needling GV20, GV26 and Yintang in SAMP8 mice by acupuncture for 15 days can significantly improve the spatial learning and memory ability of model mice, and reduce Aβ accumulation in the cerebral cortex (Jiang et al., 2019). Additionally, acupuncture combined with donepezil has more prominent effects on both cognitive performance and Aβ contents in SAMP8 mice.

#### Energy Metabolism

The brain consumes the most energy in the body. As a consequence, glucose metabolism is especially important for maintaining the normal function of the brain, especially cognitive-related functions. Acupuncture improved the learning and memory abilities of AD rats by needling GV24 and bilateral GB13 acupoints (Cui et al., 2018). Furthermore, positron emission tomography (PET) study showed that acupuncture by needling in AD rats could significantly increase the rate of glucose metabolism in the hypothalamus, thalamus, and brain stem. Similarly, acupuncture could increase cerebral glucose metabolism and ATP production through needling GV14 and BL23 in AD mice (Dong et al., 2015). After stimulation of GV14 and BL23 in AD mice, ATP levels in the cortex and hippocampus were significantly increased by up-regulating Sirtuin1 (SIRT1) and peroxisome proliferator-activated receptor γ coactivator 1-α (PGC1-α) in the cortex and hippocampus, promoting mitochondrial biosynthesis and energy metabolism. A PET study revealed that stimulating at ST36 in D-galactose injection-induced AD rat could elevate the glucose metabolism in the left olfactory cortex and bilateral amygdaloid bodies (Lu et al., 2014). Furthermore, stimulating HT7 with acupuncture is also considered to be an effective treatment for cognitive impairment in AD. Stimulating HT7 of D-galactose-induced AD rats can significantly increase the glucose metabolism rate in the cortex and hippocampus and ameliorate cognitive impairment (Lai et al., 2016).

### Imaging-Based Research on Acupuncture Treatment for AD

In recent years, functional brain imaging techniques, including fMRI and PET, have been widely used to evaluate and investigate the mechanisms of acupuncture for AD treatment. In a fMRI study, needling at HT7 in healthy young participants activated some key cognitive function related cerebral regions in the right postcentral gyrus of the frontal lobe and left inferior frontal gyrus (Chen et al., 2008). There is a low cerebral blood flow (CBF) in the brain of AD patients. By acupuncture stimulation at HT 7, CBF increased in AD rats in fMRI assay. ^18^F-fludeoxyglucose (FDG)-PET study revealed that acupuncture at HT 7 could increase the cerebral glucose metabolic rate in the hippocampus, thalamus, hypothalamus, and frontal/temporal lobes in an AD rat model (Lai et al., 2016).

A fMRI study revealed that electro-acupuncture (EA) at GV 20 induced increased regional hemodynamic (ReHo) changes in several regions associated with cognition and emotion, including the orbital frontal cortex (OFC), middle cingulate cortex (MCC), precentral cortex, and precuneus (preCUN; Yu et al., 2019). After acupunctured at GV 20, several cerebral regions closely related to AD, including the caudate nucleus (5 min after removing the needles), parahippocampal region and hypothalamus (15 min after needle removal), functionally connected to other regions in an organized network structure (Zhang et al., 2013).

Furthermore, fMRI study showed that acupuncture stimulation at ST 36 could increase amplitude of low-frequency fluctuation (ALFF) in multiple cerebral regions in cerebral cortex associated with cognition, including frontal cortex, temporal lobe, etc. in healthy volunteers (Nierhaus et al., 2015). Glucose metabolism was increased in the hippocampus, pyriform cortex, bilateral temporal lobe, right amygdala, the left orbital cortex, etc. in an AD rat model with acupuncture stimulation at ST 36 (Lu et al., 2017).

## Parkinson’s Disease

PD is the most common motor disorder in the world which is characterized by occult onset, slow progress and high disability. The main cause of the PD is the degeneration of dopaminergic neurons in the compact part of the substantia nigra (SN) and the decrease of dopamine biosynthesis in the remaining neurons, ultimately resulting in severe striatal dopamine deficiency and the development of primary motor symptoms. The pathogenesis of PD may be related to environmental factors, immunological abnormalities, mitochondrial dysfunction and oxidative stress. Because non-pharmacological therapies can improve PD symptoms to a certain extent and enchance the quality of life, about 40% of PD patients use at least one alternative therapies, acupuncture is one of the three most popular ASM treatment for PD patients (Rajendran et al., 2001; Dong et al., 2016).

### Clinical Research on Acupuncture Treatment for PD

Chen et al. (2015) reported that combined with western medicine, acupuncture had integrated effects in reducing symptoms and signs of mind, behavior, mood, complications of therapy and depression in PD patients either short-term (18 weeks) or long-term (36 weeks) treatment. In another prospective randomized study, researchers found that the gait of PD patients improved significantly after 3 weeks of EA treatment (Lei et al., 2016). Rather clinical reports have found that acupuncture can improve complications caused by PD, such as lower urinary tract symptoms (Kim et al., 2018), fatigue (Corbin et al., 2016; Kong et al., 2018), rigidity and balance (Toosizadeh et al., 2015), swallowing reflex latency (Fukuda et al., 2016), sleep disorders (Landgren et al., 2011), and so on.

In other clinical studies, bee venom acupuncture have been reported to significantly reducing PD symptoms (Doo et al., 2015), improving PD rating scale including total score, the Unified Parkinson’s Disease Rating Scale (UPDRS) motor scores parts II and III individually, as well as the berg balance scale, and the 30 min walking time (Cho et al., 2012), although the safety of bee venom acupuncture is uncertain. In a clinical study on evaluation of ASM for PD for 6-months, the researchers found that the Beck Depression Inventory (BDI) and the PD Questionnaire (PDQ-39) total score after acupoints such as St 42 (Chongyang) to Sp 3 (Taibai), LI 11 (Quchi), LI 15 (Jianyu), LI 20 (Yingxiang), ST 7 (Xiaguan), ST 36 (Tsu san li) treatment was improved, but the UPDRS motor scores worsened (Eng et al., 2006). A systematic review with meta-analysis included 11 RCTs with 831 subjects to assess the efficacy and safety of acupuncture combined with Madopar for PD (Liu et al., 2017). The results showed that acupuncture combined with Madopar can significantly improve the clinical effectiveness, the UPDRS II and UPDRS I–IV total summed scores, relieved gastrointestinal reactions, on-off phenomena and mental disorders but did not significantly reduce dyskinesia, compared with drug alone for the PD.

However, some RCTs and meta-studies have found that the effects of acupuncture for PD is not strong enough to be determined, and they speculated that the quality of trials was too low to draw any firm conclusions and suggested that the benefits of acupuncture may be due to non-specific effects (Cristian et al., 2005; Lee et al., 2013).

### Animal-Based Research on Acupuncture Treatment for PD

With the extensive use of acupuncture as an ASM therapy for PD, the mechanisms based on animal model of acupuncture treatment for PD were widely investigated. 1-methyl-4-phenyl-1,2,3,6-tetrahydropyridine (MPTP), 1-methyl-4-phenylpyridinium (MPP+), 6-hydroxydopamine (6-OHDA) and rotenone are widely used as inducers in animal models of PD (Andrzejewski et al., 2019; Gai et al., 2019; He et al., 2019).

#### Neurotransmitters

Yu et al. (2016) demonstrated that EA is able to improve motor function and enhance dopamine availability in 6-OHDA-lesioned PD mice. Wang et al. (2018) showed that the EA alleviated motor symptoms and up-regulated vesicular glutamate transporter 1 (VGluT1) in the ipsilateral subthalamic nucleus (STN) of hemi-parkinsonian rats. Lin et al. (2017) found that EA at the GB34 (Yang ling quan) and LR3 (Tai chong) acupoints increased the latency to fall from the accelerating rotarod and improved striatal dopamine levels in the MPTP-lesioned mouse model. In addition, EA inhibited apomorphine-induced rotational behavior and locomotor activity in the MPP+-lesioned rat model. Laser acupuncture at HT7 enhanced memory and neuron density in CA3 and dentate gyrus and laser acupuncture also decreased acetylcholinesterase (AChE), monoamine oxidase B (MAO-B), and malondialdehyde (MDA) while increased glutathione peroxidase (GSH-Px) in the hippocampus of a 6-OHDA lesion rats (Wattanathorn and Sutalangka, 2014). With the development of protein and genomics technics recent years, proteomic analysis demonstrated that EA reversed six proteins in unlesioned and 19 proteins in lesioned motor cortex of PD model, compared to non-treatment group (Li M. et al., 2017). These targeted proteins may be involved in maintaining the balance of neurotransmitters.

#### Neuronal Survival

Further mechanism studies have confirmed that neuroprotective effects of EA *via* the activation of survival pathways of Akt (protein kinase B, PKB) and brain-derived neurotrophic factor (BDNF) in the SN region. Li M. et al. (2017) observed the proteomic changes in the motor cortex of 6-OHDA-induced PD model after EA treatment in 4 weeks. EA significantly improved spontaneous floor plane locomotion and rotarod performance. After acupuncture stimulation at SI3 or GB34 (Yang ling quan) of MPTP induced PD mice for 12 days, acupuncture treatment increased the dopaminergic neuronal survival *via* the nigrostriatal pathway, the expression of DJ-1, the activities of superoxide dismutase and catalase in the striatum (ST; Lee et al., 2018). In the latest study, researcher reported that acupuncture activates the hypothalamic melanin-concentrating hormone (MCH) biosynthesis in PD mice, which is potential of dopaminergic neuron protection *via* downstream pathways related to neuronal survival (Park et al., 2017). Jia Y. J. et al. (2017) found that EA improved the group II metabotropic glutamate receptor (mGluR2/3) protein expression and mGluR3 mRNA expression in the striatum of (6-OHDA)-lesioned rats, which may play a critical role in the pathogenesis of PD. Other studies discovered that acupuncture stimulation at GB34 and LR3 attenuated the decrease in tyrosine hydroxylase in the SN region. Further gene array analysis revealed 22 probes (10 annotated genes: Cdh1, Itih2, Mpzl2, Rdh9, Serping1, Slc6a13, Slc6a20a, Slc6a4, Tph2, and Ucma) that were up-regulated while 17 probes (two annotated genes: 4921530L21Rik and Gm13931) that were down-regulated in MPTP animals (Yeo et al., 2015).

A systematic review of acupuncture treatment for PD in animal models by retrieving 57 articles suggest that acupuncture is an effective treatment for animal PD models, but there is insufficient evidence to determine whether sex differences exist (Lee et al., 2016). But in other studies, the results are completely contradictory, Yang et al.’s (2017) acupuncture stimulation at GB34 (Yang ling quan) in PD animal model for 5 days. Acupuncture treatment did not attenuate tyrosine hydroxylase-immunoreactive neuronal death, depletion of striatal dopamine levels, or reduced striatal tyrosine hydroxylase expression (Yang et al., 2017).

#### α-Synuclein

Stimulating at the acupoint of Yang ling quan (GB34) promoted the autophagic clearance of α-synuclein (α-syn) in PD mouse model. Further research discovered that acupuncture promotes mammalian target of rapamycin (mTOR) independent autophagic clearance of aggregation-prone proteins *in vivo* experiments (Tian et al., 2016).

#### Neuroinflammation

Furthermore, EA improved the neuro-inflammatory and motor phenotypes of mutant α-synuclein (α-syn) protein (A53T) mice multiple motor tests (Deng et al., 2015). Further study showed that EA suppressed tumor necrosis factor-α (TNF-α) and interleukin-1 β (IL-1β) in the striatum and midbrain. Similarly, bee venom acupuncture therapy (BVA) suppressed TNF-α and IL-1β in PD animal (Khalil et al., 2015). Both Deng et al. (2015) and Lv et al. (2015) found that EA improved nuclear factor-E2-related factor-2 (Nrf2), and Nrf2-regulated antioxidant enzymes in PD model.

### Imaging-Based Research on Acupuncture Treatment for PD

The levodopa combined scalp EA group significantly increased regional CBF (rCBF) in a 5-week RCT by applying single photon emission computed tomography (SPECT) measures of ^99m^Tc ethyl cysteinate dimer (^99m^Tc-ECD) and ^99m^Tc-TRODAT-4 (Huang et al., 2010). Chae et al. (2009) observed the changes of patient brain function after acupuncture in GB34 Yang ling quan through fMRI. By calculating the blood-oxygen-level dependent (BOLD), the researchers found that acupuncture might facilitate improvement in the motor functioning of patients with PD *via* the basal ganglia-thalamocortical circuit (Yeo et al., 2014). Yeo et al. (2014) also discovered that acupuncture stimulation increased neural responses in regions including the SN, caudate, thalamus, and putamen by using fMRI. EA treatment could alter neuronal activity in the striatum, primary motor cortex (M1), cingulate gyrus and global pallidus externa (GPe) in the ipsilateral hemisphere of MPTP-lesioned PD monkey in pharmacological MRI (phMRI; Zhang et al., 2018).

## Author Contributions

XG and TM collected the literature and wrote the article. TM proofread the manuscript.

## Conflict of Interest Statement

The authors declare that the research was conducted in the absence of any commercial or financial relationships that could be construed as a potential conflict of interest.
